# Methylation of SLFN11 promotes gastric cancer growth and increases gastric cancer cell resistance to cisplatin

**DOI:** 10.7150/jca.32511

**Published:** 2019-10-15

**Authors:** Yaojun Peng, Li Wang, Liangliang Wu, Ling Zhang, Guangjun Nie, Mingzhou Guo

**Affiliations:** 1 Department of Gastroenterology & Hepatology, Chinese PLA General Hospital, #28 Fuxing Road, Beijing 100853, China; 2 Department of Surgery, The Affiliated Cancer Hospital of Zhengzhou University, #127 Dongming Road, Zhengzhou, Henan Province 450008, China.; 3 Department of Oncology, Chinese PLA General Hospital, #28 Fuxing Road, Beijing 100853, China.; 4 CAS Key Laboratory for Biomedical Effects of Nanomaterials and Nanosafety, North Road No. 1, Zhongguancun, Beijing, 100190, China.

**Keywords:** SLFN11, gastric cancer, epigenetics, methylation, DNA damage repair, cisplatin

## Abstract

**Background and Aim:** Human SLFN11 gene encodes a protein with structural similarity to RNA helicases, which was reported to sensitize cancer cells to DNA-damaging agents. This study explored the epigenetic regulation and mechanism of SLFN11 in human gastric cancer.

**Methods:** Eight human gastric cancer cell lines and 201 cases of primary gastric cancer were analyzed. Methylation specific PCR, flow cytometry, xenograft mouse model and siRNA technique were employed.

**Results:** SLFN11 was methylated in 29.9% (60/201) of primary gastric cancer. The expression of SLFN11 was regulated by promoter region methylation. Methylation of SLFN11 was significantly associated with tumor size (p < 0.05). SLFN11 suppressed gastric cancer growth both *in vitro* and *in vivo* and enhanced the ability of cisplatin to induce S-phrase arrest and apoptosis in gastric cancer cells.

**Conclusions:** SLFN11 is frequently methylated in human gastric cancer, and its expression is regulated by promoter region methylation. Our results demonstrate that SLFN11 is a tumor suppressor in human gastric cancer, and methylation of SLFN11 may serve as a cisplatin resistant marker in human gastric cancer.

## Introduction

Gastric cancer (GC) is the third most lethal cancer worldwide^1^. *H. pylori* (HP) infection is strongly associated with GC^2^. HP triggers the DNA damage response and immune response crosstalk promoting a vicious cycle of DNA damage and persistent inflammation that fuels tumorigenesis^3^. Although most GCs are sporadic, aggregation within families occurs in roughly 10% of cases. Truly hereditary cases are thought to account for 1-3% of GC, including at least three main syndromes: hereditary diffuse gastric cancer, gastric adenocarcinoma and proximal polyposis of the stomach, and familial intestinal gastric cancer^4^, ^5^. Hereditary diffuse gastric cancer was recognized to be caused by inherited CDH1 mutations^6^.

Hereditary nonpolyposis colorectal cancer (CRC), also referred to as the Lynch syndrome, is the most common form of hereditary colorectal cancer. Lynch syndrome has a molecular phenotype of microsatellite instability that is caused by a germ-line mutation in any of the mismatch repair genes MLH1, MSH2, MSH6, PMS1, PMS2, or EPCAM^7^. The frequency of gastric cancer in carriers of Lynch syndrome mutations has been estimated at 1.6%, and it mainly happens to intestinal-type of Lauren classification^8^. The frequency of gastric cancer in families carrying TP53 mutations ranges from 1.8% to 4.9%^9^. A meta-analysis of more than 30 studies showed that the relative risk of GC in BRCA1 or BRCA2 carriers is 1.69%, which is higher than the relative risk for pancreatic, prostate, and colorectal cancer^10^. In microsatellite unstable sporadic GC, the mismatch repair defect is mainly caused by MLH1 promoter region hypermethylation^11^.

Human cells and their genome are under constant attack by DNA-damaging agents, resulting in tens of thousands of DNA lesions daily 12. The cells respond to DNA lesions by activating a complex mechanism, which is named DNA damage response (DDR)^13^. Defects of DDR may induce carcinogenesis^14^. Schlafen (Slfn) (from the word schlafen, which in German means sleeping) genes were originally identified during screening for growth regulatory genes^15^-^18^. To date, 10 mouse (Slfn1, 1L, 2, 3, 4, 5, 8, 9, 10, and 14) and 6 human (Slfn5, 11, 12, 12L, 13, and 14) Slfn genes have been identified^15^-^18^. SLFN11 was discovered by bioinformatics analyses of cancer cell databases as a causal and dominant genomic determinant of response to widely used anticancer drugs, including topoisomerase Ⅰ (camptothecin) /Ⅱ inhibitors (etoposide), alkylating agents (cisplatin and carboplatin) and DNA synthesis inhibitors (gemcitabine)^19^. Our previous study found that SLFN11 is frequently methylated in CRC, and methylation of SLFN11 reduced the sensitivity of CRC cells to cisplatin^20^. The epigenetic regulation and function of SLFN11 in human GC remain to be elucidated.

## Materials & Methods

### Human tissue samples & cell lines

A total of 201 cases of GC samples and 8 cases of normal gastric mucosa from noncancerous patients were collected from the Chinese PLA General Hospital. In addition, eight GC cell lines were involved in this study. The study was approved by the Chinese PLA General Hospital's Institutional Review Board.

### 5-aza-2′-deoxycytidine treatment

GC cells were treated with 2 μM 5-aza-2′-deoxycytidine (5-AZA, Sigma, MO, USA) for 96 h.

### RNA isolation & semi-quantitative RT-PCR

Total RNA was isolated by Trizol reagent (Invitrogen, Carlsbad, USA). PCR primers for SLFN11 and GAPDH are listed in Table 1.

### Bisulfite modification, methylation-specific PCR (MSP) and bisulfite sequencing (BSSQ)

Bisulfite treatment was carried out as previously described^21^. BSSQ primers encompassed a 268 bp (-718 to -451 bp) region upstream of the transcription start site (TSS) of SLFN11. MSP and BSSQ primers are listed in Table 1. The thermal cycling conditions were described previously^20^.

### Construction of human full length SLFN11 cDNA lentiviral expression vector & transient expression vector

The human full length SLFN11 cDNA (NM_001104587) was cloned into the PCDH (pCDH-CMV-MCS-EF1-Puro) lentiviral plasmid. Lentivirus was packaged by transfection into 293T cells using Lipofectamine 3000 (Invitrogen, CA, USA). GC cells stably re-expressing SLFN11 were established by infection with viral supernatant and subsequent selection with puromycin (2 μg/ml for 2 weeks). For transient transfection, SLFN11 cDNA was cloned into the pcDNA3.1(+) plasmid (Era Biotech, Shanghai, China), and transfection was performed using Lipofectamine 3000 (Invitrogen, CA, USA).

### siRNA knockdown technique

The sequences of three siRNAs targeting SLFN11 and the RNAi negative control duplex are listed in Table 1 (Gene Pharma Co, Shanghai, China). The efficacy of the three siRNAs was determined by transfection into SLFN11 highly expressing NUGC3 cells using Lipofectamine RNAiMAX (Invitrogen, CA, USA). The most effective siRNA, Si1726, was selected for the subsequent study (Figure 5C).

### Cell viability assay

Cell viability were measured by the methyl thiazolyl tetrazolium assay (MTT, KeyGEN Biotech, Nanjing, China) at 0, 24, 48 and 72 h for SNU16 and MGC803 cells, and an additional 24 h (up to 96 h) for NUGC3 cell viability.

### Colony formation assay

GC cells were seeded into 6-well culture plates at a density of 800 cells per well in triplicate. After 2 weeks, cells were fixed with 75% ethanol for 30 min and stained with 0.2% crystal violet (Beyotime, Nanjing, China).

### Colony formation assay treated with cisplatin

GC cells were plated onto 6-well plates at a total of 1000 cells per well. After overnight incubation, cells were treated with cisplatin (1 μM) for 24 h and maintained in drug-free medium for an additional 2 weeks until visible colonies were evident. Cells were stained with 0.2% crystal violet.

### Flow cytometry for cell cycle & apoptosis

For cell cycle analysis, cells were serum starved 12 hours for synchronization, re-stimulated with complete medium supplemented with 2 μM cisplatin for 24 h, fixed with 70% ethanol, and prepared for flow cytometry analysis using the Cell Cycle Detection Kit (KeyGenBiotech, Nanjing, China).

For apoptosis analysis, GC cells were seeded in 6-well plates at a density of 1.5 × 106 cells/ml. Cells were treated with a concentration of 2 μM cisplatin for 24 h, harvested using 0.2% trypsin without ethylene diamine-tetraacetic acid (EDTA), stained according to manufacturer's instructions of the Annexin V-FITC/PI Apoptosis Detection Kit (KeyGen Biotechnology, China).

### Immunohistochemistry (IHC)

Immunohistochemistry (IHC) was performed in MGC803 cell xenografts. Rabbit polyclonal antibody against SLFN11 (Sigma-Aldrich, St. Louis, MO, USA) was diluted at 1:50. The procedure was performed as described previously^22^, ^23^.

### Western blot

Western blotting was performed as described previously^20^. Antibodies were listed as follow: rabbit anti-SLFN11 (Santa Cruz Biotechnology, CA, USA), rabbit anti-Bax, rabbit anti-Bcl2, rabbit anti-caspase3, rabbit anti-cleaved-caspase3 and mouse anti-β-actin (Proteintech, IL, USA).

### SLFN11 unexpressed & re-expressed MGC803 cell xenograft mouse model

SLFN11 unexpressed or stably re-expressed BGC823 cells (1 × 106) were suspended in 0.1 ml phosphate buffer saline (PBS) and injected subcutaneously into the dorsal left side of 4-week-old male Balb/c nude mice (5 per group). The diameter of the tumors was measured every 3 days. Tumor volume (mm3) was estimated by the following formula: tumor volume = (length) × (width)2/2. Mice were sacrificed at the 21st day after inoculation, and tumor weights were measured after dissection. All procedures were approved by the Animal Ethics Committee of the Chinese PLA General Hospital.

### Statistical analysis

The Illumina Infinium Human Methylation 450K (HM450K) data and mRNA expression data of SLFN11 were downloaded from The Cancer Genome Atlas (TCGA) database (https://cancergenome.nih.gov/, 05/12/2018). Statistical analysis was performed using SPSS 17.0 software (SPSS Inc., Chicago, IL). The association of SLFN11 methylation and different clinical factors was evaluated using chi-square test. The association of SLFN11 methylation and clinical factors were estimated by univariate and multivariate binary logistic regression. The student's t-test was applied to determine the statistical significance between two experimental groups. The association of DNA methylation in different CpG site and the levels of SLFN11 RNA expression were analyzed by Pearson's correlation coefficient. Two-sided tests were used to determine significance, and p < 0.05 was considered statistically significant.

## Results

### The expression of SLFN11 is regulated by promoter region methylation in human GC

**Figure 1:**
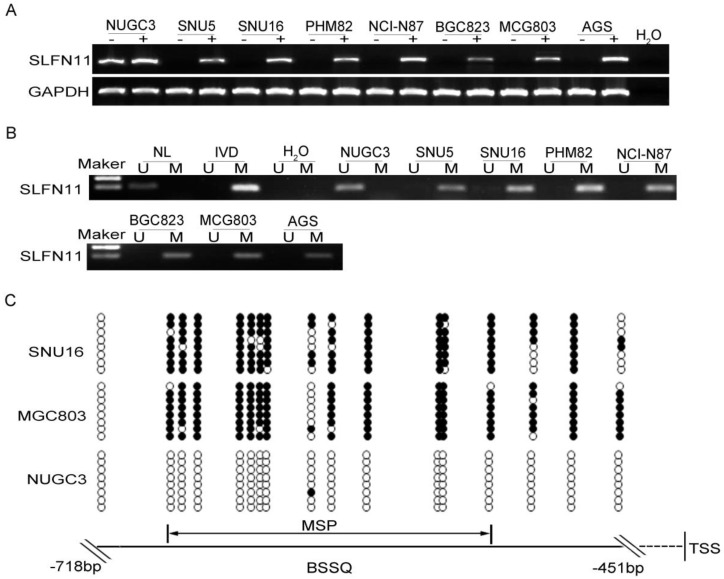
** Expression of SLFN11 is silenced by DNA methylation in GC cell lines. (A)** Expression of SLFN11 was analyzed by RT-PCR in GC cell lines in the absence (-) or presence of 2 μM 5-AZA (+) for 96 h. **(B)** Methylation status of SLFN11 detected by MSP in GC cell lines. NL: normal lymphocyte DNA; IVD: *in vitro* methylated DNA; M: methylated alleles; U: unmethylated alleles. **(C)** Bisulfite sequencing of SLFN11 was performed in SNU16, MGC803 and NUGC3 cell lines. The region of CpG islands studied by MSP was indicated by a double-headed arrow. Filled circles represent methylated CpG sites within the SLFN11 CpG islands and open circles denote unmethylated CpG sites. Bisulfite sequencing focused on a 268 bp (-718 to -451 bp) CpG islands upstream of the SLFN11 TSS.

The expression levels of SLFN11 in GC cell lines were examined by semi-quantitative RT-PCR, and promoter region methylation was detected by MSP. SLFN11 was highly expressed and unmethylated in NUGC3 cells. SLFN11 was unexpressed and completely methylated in SNU5, SNU16, MGC803, PHM82, NCI-N87, BGC823 and AGS cells (Figure 1A, 1B). Loss of SLFN11 expression was correlated with promoter region hypermethylation. The methylation status was validated by BSSQ in SNU16, MGC803 and NUGC3 cells (Figure 1C). Upon treatment with 5-AZA, a demethylating agent, restoration of SLFN11 expression was observed in SNU5, SNU16, MGC803, PHM82, NCI-N87, BGC823 and AGS cells, and no expression changes were detected in NUGC3 cells (Figure 1A). These results suggest that the expression of SLFN11 is regulated by promoter region methylation.

### SLFN11 is frequently methylated in human GC

**Figure 2:**
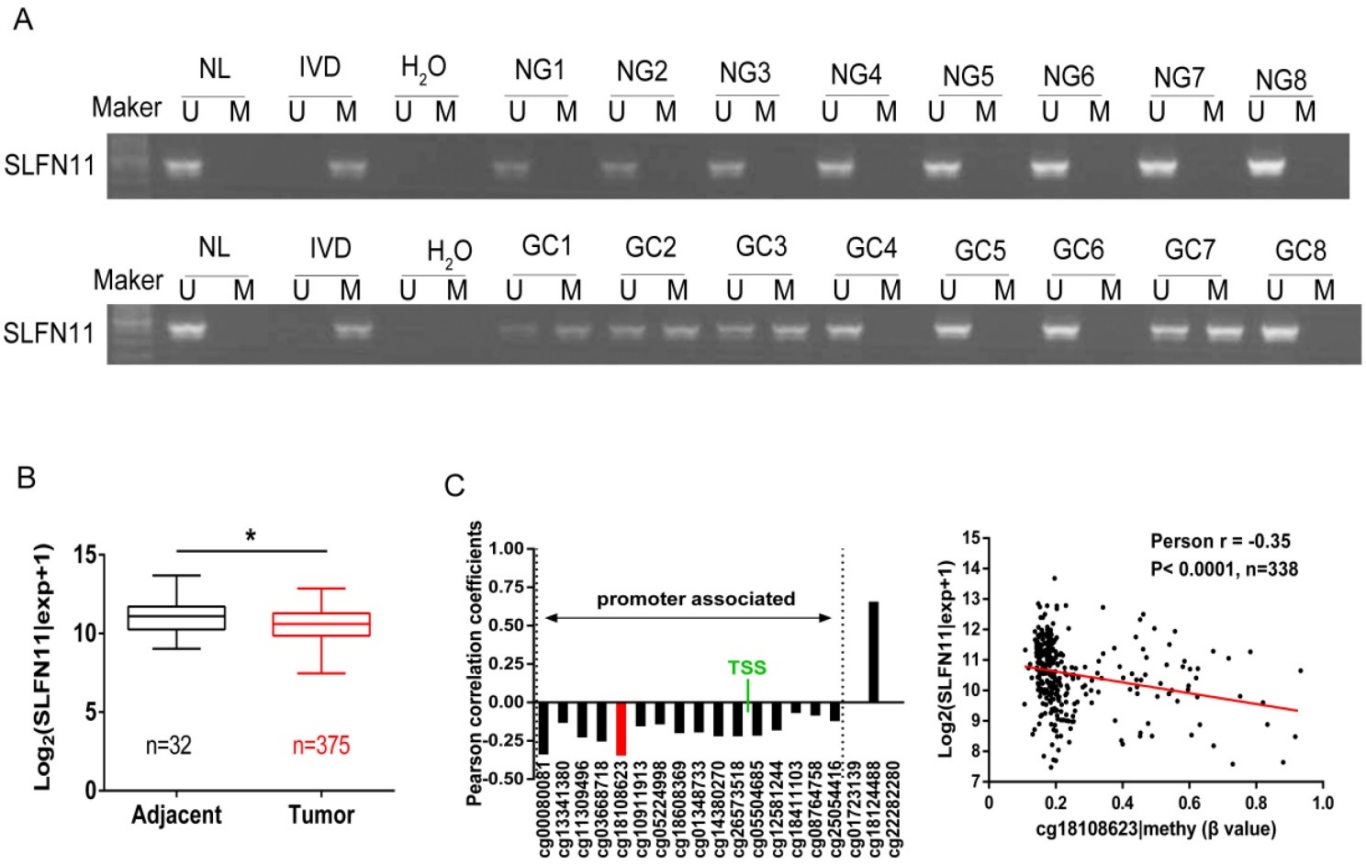
** Epigenetic inactivation of SLFN11 in primary GC. (A)** Representative MSP results of SLFN11 methylation status in normal gastric mucosa (NG) and GC tissues (GC). **(B)** TCGA data showed SLFN11 mRNA expression levels in GC tissues (n=375) and normal gastric mucosa (n=32) according to RNA-seq results. Box plots: The levels of SLFN11 expression. Horizontal lines: log_2_ (normalized counts + 1) (*p < 0.05). **(C)** The correlation of methylation of 19 CpG sites and expression of SLFN11 (left panel). The methylation status of the top promoter associated CpG site (cg18108623) was significantly negatively correlated with of SLFN11 expression in 338 cases of GC (Pearson r = -0.35, p < 0.001).

The methylation status of SLFN11 in human primary GC was detected by MSP. SLFN11 was methylated in 29.9% (60/201) of GC, while no methylation was found in eight cases of normal gastric mucosa from noncancerous patients (Figure 2A). In 128 cases of tumor diameter ≥ 5cm of patients, SLFN11 was methylated in 35.16% (45 of 128). In 73 cases of tumor diameter < 5cm of patients, SLFN11 was methylated in 20.55% (15 of 73). The methylation rate of SLFN11 is significantly higher in diameter ≥ 5cm tumors than in diameter < 5cm tumors (p < 0.05, Table 2). No association was found between SLFN11 methylation and age, gender, tumor differentiation, TNM stage and vessel invasion (all p > 0.05, Table 2). SLFN11 methylation was associated with tumor diameter by univariate logistic regression analysis (p < 0.05, Table 3). According to multivariate binary logistic regression analysis, SLFN11 methylation is not associated with tumor size after adjusting for age, gender, tumor differentiation, TNM stage and vessel invasion (all p > 0.05, table 3).

To further validate that the expression of SLFN11 was regulated by promoter region methylation in primary GC, TCGA database was employed. SLFN11 mRNA data were obtained from 375 cases of GC samples and 32 cases of normal gastric tissue samples according to RNA-Seq. The expression levels of SLFN11 were significantly lower in GC samples compared to normal gastric tissue samples (p < 0.05, Figure 2B). Available data for matched SLFN11 expression and methylation were obtained from 338 cases of GC samples. In total, 19 CpGs were analyzed for SLFN11 gene methylation by Illumina Infinium HM450K assay. The correlation between SLFN11 gene expression and methylation status of 19 CpGs was shown in Figure S1. The expression of SLFN11 was inversely associated with the methylation status of 16 CpGs (Figure 2C, S1), no association was found between gene expression and methylation status in two CpGs in gene body region (cg01723139, cg22282280), and the expression was positively associated with DNA methylation in one CpG in gene body region (cg18124488). These results further suggest that the expression of SLFN11 is regulated by promoter region methylation.

### Restoration of SLFN11 expression suppresses proliferation of human GC cells

**Figure 3:**
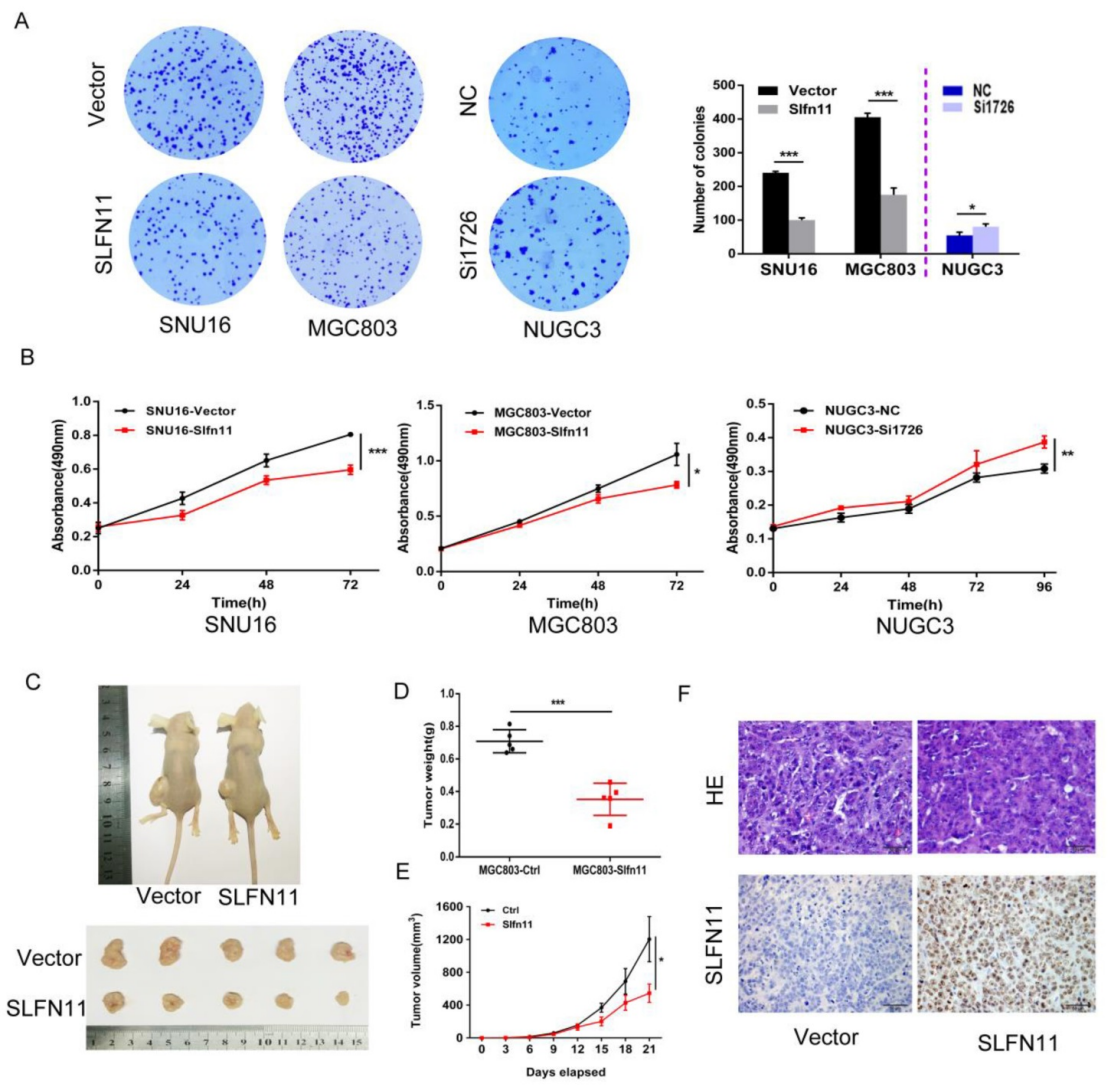
** SLFN11 suppresses GC cell proliferation *in vitro* and *in vivo*. (A**) Representative results of colony formation in SNU16 and MGC803 cells before and after stably restoration of SLFN11 expression, and in NUGC3 cells before and after knockdown of SLFN11. The experiment was repeated three-times. **(B)** Growth curves demonstrate the effects of SLFN11 on cell proliferation as measured by the MTT assay in SNU16 and MGC803 cells for 72 h before and after transient restoration of SLFN11 expression, and in NUCG3 cells for 96 h before and after knockdown of SLFN11. The experiment was repeated three times. **(C)**Representative images of xenograft tumors from SLFN11 unexpressed and stably re-expressed MGC803 cells. **(D)** Average weight of xenograft tumors in nude mice with or without SLFN11 re-expression. **(E)** Tumor growth curves of xenograft tumors with or without SLFN11 re-expression. **(F)** Representative images of H&E and SLFN11 immunohistochemistry staining in MGC803 cell xenograft tumors. ***p < 0.001, **p < 0.01*p < 0.05

SNU16 and MGC803 cells were stably re-expressed of SLFN11 via lentiviral infection and colony formation assays were performed to evaluate the effect of SLFN11 on clonogenicity. The colony numbers before and after restoration of SLFN11 expression were 240 ± 4 versus 100 ± 7 in SNU16 cells and 406 ± 12 versus 175 ± 20 (both p< 0.001) in MGC803 cells. The colony numbers before and after siRNA knockdown of SLFN11 were 55 ± 10 compared to 81 ± 8 (p < 0.05, Figure 3A). These differences suggest that colony formation was suppressed by SLFN11. To further evaluate the effects of SLFN11 on cell proliferation, SNU16 and MGC803 cells were transient transfected with SLFN11 plasmid or control vector, and cell viability was detected by MTT assays. The OD values before and after restoration of SLFN11 expression were 0.805 ± 0.006 versus 0.596 ± 0.028 in SNU16 cells and 1.058 ± 0.100 versus 0.784 ± 0.031 in MGC803 cells (both p < 0.05, Figure 3B). The OD values were 0.309 ± 0.013 versus 0.387 ± 0.018 (p < 0.01) before and after knockdown of SLFN11 in NUGC3 cells (Figure 3B). These data indicate that cell viability decreased after restoration of SLFN11 expression in SNU16 and MGC803 cells and increased after knockdown of SLFN11 in NUGC3 cells. No obvious morphological changes were driven by SLFN11 (Figure S2). Collectively, these results suggest that SLFN11 suppresses proliferation of GC cells.

### SLFN11 suppresses human GC cell xenograft growth in mice

To further investigate the effects of SLFN11 on human GC, SLFN11 unexpressed and re-expressed MGC803 cell xenograft mouse models were employed (Figure 3C). The xenograft tumor weight was 0.709 ± 0.071 g in SLFN11 unexpressed MGC803 cell tumors and 0.352 ± 0.099 g in SLFN11 re-expressed MGC803 cell tumors. The tumor weights were significantly reduced in SLFN11 re-expressed MGC803 cell tumors (P < 0.001, Figure 3D). The tumor volume was 1204.8 ± 616.3 mm3 in SLFN11 unexpressed MGC803 cell xenografts and 544.9 ± 247.6 mm3 in SLFN11 re-expressed MGC803 cell xenografts. The tumor volumes were significantly smaller in SLFN11 re-expressed MGC803 cells compared to SLFN11 unexpressed MGC803 cells (P < 0.05, Figure 3E). Re-expression of SLFN11 was validated by IHC staining in re-expressed MGC803 cell xenografts (Figure 3F). These results suggest that SLFN11 suppresses human GC cell growth in vivo.

### SLFN11 sensitizes human GC cells to cisplatin

**Figure 4:**
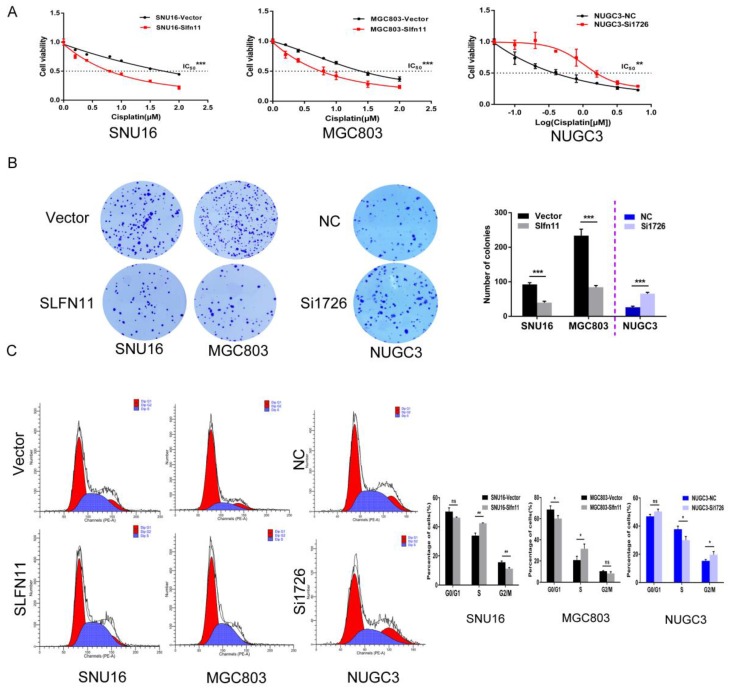
** SLFN11 enhances chemo-sensitivity of GC cells to cisplatin* in vitro*. (A)** Cell viability determined by MTT assays following exposure to cisplatin for 72h. **(B)** Colony formation under treatment with 1 μM cisplatin in SNU16 and MGC803 cells before and after stably re-expression of SLFN11, and in NUGC3 cells before and after knockdown of SLFN11. The experiment was repeated three times.** (C)** Cell phase distribution was evaluated by flow cytometry under treatment of cisplatin before and after stably restoration of SLFN11 expression in SNU16 and MGC803 cells and before and after knockdown of SLFN11 in NUGC3 cells. ***p < 0.001, **p < 0.01, *p < 0.05, ns: no significance

Recently, SLFN11 has been identified as a causal and dominant genomic determinant of response to a range of DNA damaging agents in a variety of human cancers^19^, ^20^, ^24^-^27^. To explore whether SLFN11 could sensitized GC cells to cisplatin, a DNA damaging agent, MTT assay was employed. The IC50 values of cisplatin for 72h were 1.920 ± 0.083 versus 0.750 ± 0.056 μM and 1.404 ± 0.057 versus 0.721 ± 0.043 μM before and after stable re-expression of SLFN11 in SNU16 and MGC803 cells, respectively. The IC50 values were significantly reduced after re-expression of SLFN11 (both p < 0.001, Figure 4A). The IC50 values were 0.504 ± 0.131 versus 2.028 ± 0.305 μM in NUGC3 cells before and after knockdown of SLFN11. The IC50 value was significantly increased after knockdown of SLFN11 (p < 0.01, Figure 4A). These results suggest that SLFN11 sensitizes GC cells to cisplatin.

The sensitizing effects of SLFN11 on GC cells were also determined in the long-term colony formation assay. Under treatment with 1 μM of cisplatin, the numbers of colonies before and after stable re-expression of SLFN11 were 92 ± 5 versus 40 ± 4 in SNU16 cells and 234 ± 19 versus 84 ± 5 in MGC803 cells. The colony numbers were significantly reduced after re-expression of SLFN11 in SNU16 and MGC803 cells (both p < 0.001, Figure 4B). Upon treatment with 1 μM of cisplatin, the number of colonies was 26 ± 3 versus 66 ± 4 in NUGC3 cells before and after knockdown of SLFN11. The number of colonies increased significantly after knockdown of SLFN11 in NUGC3 cells (p < 0.001, Figure 4B). These results further indicate that SLFN11 sensitizes GC cells to cisplatin.

### SLFN11 enhanced the ability of cisplatin to induce S-phase arrest in human GC cells

SLFN11 was recently reported to block stressed replication forks by inhibiting RPA binding to nascent DNA^28^. To explore the possible effects of SLFN11 and cisplatin synergizing to induce S-phase arrest in gastric cancer, SLFN11 unexpressed and stably re-expressed GC cells were treated with cisplatin (2 μM, 24h). Cell phase distribution was evaluated by flow cytometry. The cell phase distributions in SLFN11 unexpressed and re-expressed SNU16 cells were as follows: G0/G1 phase, 50.50 ± 2.60% versus 46.29 ± 0.43% (p = 0.051); S phase, 33.93 ± 1.75% versus 42.40 ± 0.33% (p = 0.0012); G2/M phase, 15.57 ± 0.85% versus 11.31 ± 0.68% (p = 0.0025). The percentage of cells in S phase increased significantly after re-expression of SLFN11 in SNU16 cells (p < 0.01, Figure 4C). The cell phase distributions in SLFN11 unexpressed and re-expressed MGC803 cells were as follows: G0/G1 phase, 68.65 ± 3.40% versus 60.07 ± 2.84% (p = 0.029); S phase, 20.77 ± 3.63% versus 31.53 ± 4.42% (p = 0.031); and G2/M phase, 10.52 ± 0.43% versus 8.37 ± 1.60% (p = 0.089). The percentage of cells in S phase increased significantly after re-expression of SLFN11 in MGC803 cells under cisplatin treatment (p < 0.05, Figure 4C). To further validate the synergistic effect of SLFN11 and cisplatin, SLFN11 was knocked down by siRNA in NUGC3 cells under the treatment of cisplatin. The cell phase distributions before and after knockdown of SLFN11 were as follows: G0/G1 phase,46.95 ± 1.45% versus 50.37 ± 1.63% (p = 0.053); S phase, 37.79 ± 2.20% versus 29.98 ± 2.62% (p = 0.017); and G2/M phase, 15.27 ± 0.93% versus 19.65 ± 2.18% (p = 0.033). The percentage of cells in S phase was reduced significantly after knockdown of SLFN11 in NUGC3 cells under the treatment of cisplatin (p < 0.05, Figure 4C). These results suggest that SLFN11 synergized with cisplatin to induce S-phase arrest in GC cells.

### SLFN11 increased apoptosis in human GC cells induced by cisplatin

**Figure 5:**
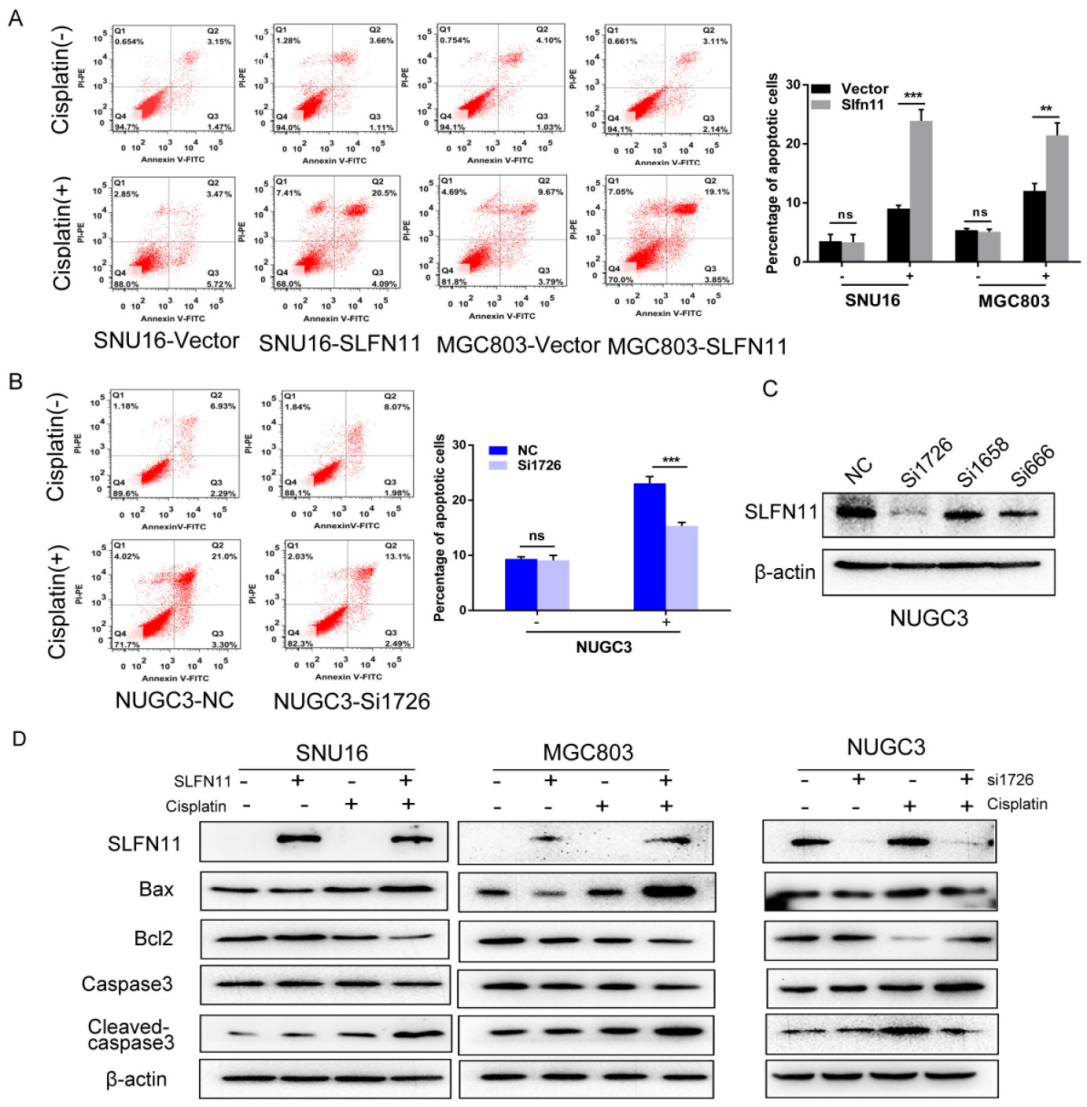
** SLFN11 increased apoptosis in human GC cells induced by cisplatin (A)** Apoptosis was detected and quantified by annexin V-FITC and propidium iodide staining in SNU16 and MGC803 cells. The experiment was repeated three times. **(B)** Apoptosis was detected and quantified by annexin V-FITC and propidium iodide staining in NUGC3 cells. **(C)** The effect of siRNAs on SLFN11 expression in NUGC3 cells. **(D)** Expression levels of SLFN11 and apoptosis related proteins, including Bax, Bcl2, caspase3, and cleaved-caspase3, were detected by Western blot. β-actin was used as a control. ***p < 0.001, **p < 0.01, ns: no significance

To further validate role of SLFN11 sensitizing cisplatin, the effects of SLFN11 on apoptosis were analyzed. Without cisplatin treatment, the percentages of apoptotic cells were 3.51 ± 1.16% versus 3.31 ± 1.31% before and after re-expression of SLFN11 stably in SNU16 cells (p> 0.05) and 5.38 ± 0.29% versus 5.12 ± 0.42% in MGC803 cells (p > 0.05) (Figure 5A). No significant changes were observed after re-expression of SLFN11 in GC cells. In SLFN11 highly expressed NUGC3 cells, the percentages of apoptotic cells were 9.37 ± 0.37% versus 9.10 ± 0.92% before and after knockdown of SLFN11 (p >0.05) (Figure 5B). No significant changes were observed knockdown of SLFN11. Upon treatment with 2 μM cisplatin, the percentages of apoptotic cells before and after stable re-expression of SLFN11 were 8.90 ± 0.38% versus 23.90 ± 1.92% (p < 0.001) in SNU16 cells and 12.04 ± 1.24% versus 21.47 ± 2.08% in MGC803 cells (p < 0.01) (Figure 5A). In NUGC3 cells, the percentages of apoptotic cells were 23.08 ± 1.23% versus 15.37 ± 0.61% before and after knockdown of SLFN11 (p < 0.001) (Figure 5B). The amount of apoptosis induced by cisplatin treatment significantly increased after re-expression of SLFN11 and significantly decreased after knockdown of SLFN11. Apoptosis related proteins, Bax, Bcl2, caspase3, and cleaved-caspase3, were detected by Western blot. Upon treatment with cisplatin, the levels of pro-apoptotic proteins, cleaved caspase-3 and Bax, increased after re-expression of SLFN11 in SNU16 and MGC803 cells and decreased after knockdown of SLFN11 expression in NUGC3 cells (Figure 5D). The changes in anti-apoptotic protein, Bcl2, showed opposite trends to the pro-apoptotic proteins in these cells (Figure 5D). These results further suggest that SLFN11 sensitizes GC cells to cisplatin.

## Discussion

Given the impact that the DDR can have on both disease development and response to treatment, it seems reasonable to consider categorizing tumors according to their DNA repair defects and to use this information to personalize treatment^29^. Silencing of DDR genes by promoter region hypermethylation was frequently found in human gastric cancers^30^, ^31^. In the clinic, functional biomarkers of the DDR-pathway should be identified before treatment with a DNA-damaging agent. SLFN11 is a nuclear protein and its expression is causally associated with the activity of DNA-damaging agents in human cancer^19^. Our previous study found that SLFN11 is frequently methylated in human CRC and SLFN11 methylation reduced sensitivity of CRC to cisplatin^20^. In this study, we found that SLFN11 is methylated in 29.9% of human GC, and the expression of SLFN11 is regulated by promoter region methylation. Methylation of SLFN11 is significantly associated with tumor size. SLFN11 suppressed GC cell growth both in vitro and in vivo. These results suggest that SLFN11 is a tumor suppressor in human GC, and SLFN11 methylation is a potential GC detection marker.

DNA-damage checkpoints occur throughout the cell cycle. If DNA damage occurs during S-phase, the intra-S-phase checkpoint is activated blocking further replication^32^. Cisplatin is reported to cause replication arrest by DNA crosslinking in the S-phase^33^. Several studies indicated that DNA synthesis continues to occur in cells that fail to divide following treatment with cisplatin^34^, suggesting that a cellular mechanism for bypassing platinum-DNA lesions may be operative. This mechanism is now known to be translesion synthesis (TLS), and the efficiency and fidelity with which cells are able to bypass DNA-platinum lesions is linked to drug sensitivity^35^.

SLFN11 was reported to block stressed replication forks by inhibiting RPA binding to nascent DNA^28^. Thus, we explored the synergistic activity of SLFN11 and cisplatin in GC cells. As expected, S-phase arrest was induced by adding cisplatin to SLFN11 re-expressed GC cells compared to SLFN11 unexpressed parental GC cells. These results suggest that SLFN11 methylation is a resistant marker for cisplatin in human GC.

In conclusion, SLFN11 is frequently methylated in human GC, and the expression of SLFN11 is regulated by promoter region methylation. Methylation of SLFN11 was significantly associated with tumor size. SLFN11 is a tumor suppressor in human GC, and methylation of SLFN11 is a cisplatin resistant marker in human GC.

## Supplementary Material

Supplementary figures and tables.

## Figures and Tables

**Table 1:** Primers and siRNA used in this study

Primers/siRNA	Sequence (5'-3')
RT-SLFN11-F	AACGCCCGATAACCTTCACA
RT-SLFN11-R	CTAAGGGGAGGCCCACTAGA
RT-GADPH-F	GACCACAGTCCATGCCATCAC
RT-GADPH-R	GTCCACCACCCTGTT GCTGTA
MSP-MF	ATTATTAGTAGCGTGACGGTTATC
MSP-MR	CGACAAATATACAAATTAAACCGCG
MSP-UF	TATATTATTAGTAGTGTGATGGTTATT
MSP-UR	ATACAACAAATATACAAATTAAACCACA
BSSQ-F	TAGAAAAGTAGAAYGTTGGTAG
BSSQ-R	CAAAAAATAAATCTTAAAAAC
siRNA#NC-sense	UUCUCCGAACGUGUCACGUTT
siRNA#NC-antisense	ACGUGACACGUUCGGAGAATT
siRNA#666-sense	GGCUGCAUGUGCUUUAUUATT
siRNA#666-antisense	UAAUAAAGCACAUGCAGCCTT
siRNA#1658-sense	GACCAGUGUACUCCAAGAATT
siRNA#1658-antisense	UUCUUGGAGUACACUGGUCTT
siRNA#1726-sense	CCAGGAUAUUUGCGAUAUATT
siRNA#1726-antisense	UAUAUCGCAAAUAUCCUGGTT

**Table 2:** Clinical factors and SLFN11 methylation in 201 cases of gastric cancer samples

Clinical factor	SLFN11 methylation status		P value
	Unmethylated n=141 (70.1%)	Methylated n=60 (29.9%)	
Age (year)			0.070
<50	32	7	
≥50	109	53	
Gender			0.243
Male	107	50	
Female	34	10	
Tumor diameter (cm)			0.030*
<5	58	15	
≥5	83	45	
Tumor differentiation			0.540
Poor	78	36	
Moderate-well	63	24	
TNM stage			0.142
I-II	48	27	
III-IV	93	33	
Vessel invasion			0.579
Negative	95	38	
Positive	46	22	

P-values are obtained from chi-squared test. Statistically significant, *: p<0.05.

**Table 3:** Binary logistic regression analysis of associated factors for SLFN11 methylation in gastric cancer samples

Clinical factor	Univariate analysis		Multivariate analysis	
	OR (95% CI)	P value	OR (95% CI)	P value
Age (<50 vs ≥50 years)	2.223 (0.921-5.366)	0.076	2.114 (0.853-5.237)	0.106
Gender (Male vs Female)	0.629 (0.288-1.374)	0.245	0.677 (0.300-1.528)	0.347
Tumor diameter (<5 vs ≥5cm)	2.096 (1.069-4.112)	0.031*	2.007 (0.996-4.044)	0.051
Tumor differentiation (poor vs moderate-well)	0.825 (0.447-1.525)	0.540	0.772 (0.405-1.470)	0.431
TNM stage (I-II vs III-IV)	0.631 (0.341-1.169)	0.143	0.575 (0.302-1.093)	0.091
Vessel invasion (negative vs positive)	1.196 (0.635-2.250)	0.580	1.185 (0.608-2.308)	0.619

## Abbreviations

GC: gastric cancer
HP: H. pylori
CRC: colorectal cancer
5-AZA: 5-aza-2′-deoxycytidine
MSP: methylation-specific PCR
BSSQ: bisulfite sequencing
GAPDH: glyceraldehyde-3-phosphatedehydrogenase
TSS: transcription start site
EDTA: ethylene diamine-tetraacetic acid
TCGA: The Cancer Genome Atlas
TLS: translesion synthesis
