# Supplementary material for: Methylation of SLFN11 promotes gastric cancer growth and increases gastric cancer cell resistance to cisplatin
Source: J Cancer. 2019 Oct 15;10(24):6124–34. doi: 10.7150/jca.32511 (PMC6856579; doi:10.7150/jca.32511)
Supplement: Supplementary file 1 — Supplementary figures and tables. [file jcav10p6124s1.pdf]

Legend of supplementary figures

Figure S1: Correlation between SLFN11 gene expression and methylation of 19 CpGs in TCGA database. In total, 19 CpGs were analyzed for SLFN11 gene methylation by Illumina Infinium HM450K assay. The expression of SLFN11 was inversely associated with the methylation status of 16 CpGs, and the expression of SLFN11 was associated with the methylation status of one CpG site (cg18124488) positively. β values of two CpGs (cg01723139 and cg22282280) in all GC samples were 0 (dots in red).

Figure S2: The effect of SLFN11 on morphology of GC cells. No obvious morphological changes were found before and after re-expression of SLFN11 in SNU16 and MGC803 cells, as well as in NUGC3 cells before and after knockdown of SLFN11 (48 hours). (Right) Western blot showing SLFN11 expression after transfection of SLFN11 plasmid vector or SLFN11 targeting siRNA. Magnification: ×200.

Figure S1

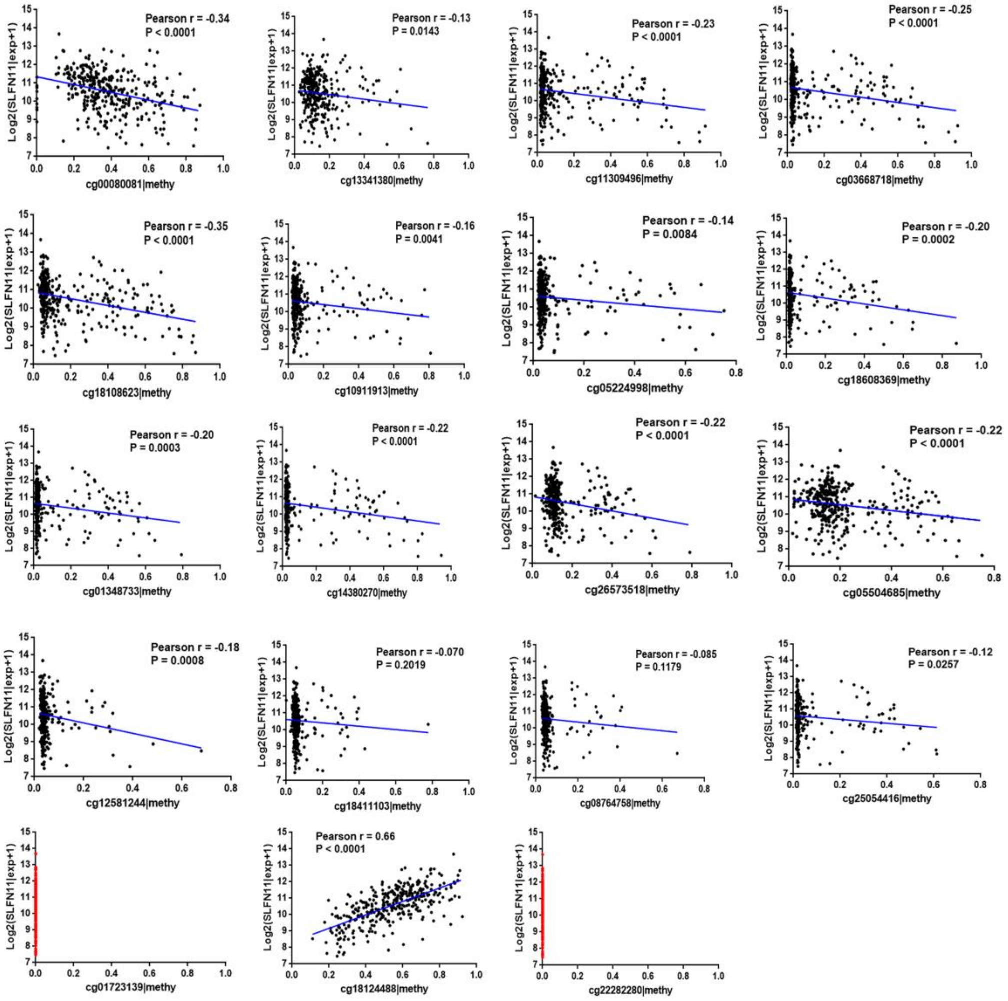
Pearson r = -0.34
P < 0.0001
cg00080081|methy
Pearson r = -0.13
P = 0.0143
cg13341380|methy
Pearson r = -0.23
P < 0.0001
cg11309496|methy
Pearson r = -0.25
P < 0.0001
cg03668718|methy
Pearson r = -0.35
P < 0.0001
cg18108623|methy
Pearson r = -0.16
P = 0.0041
cg10911913|methy
Pearson r = -0.14
P = 0.0084
cg05224998|methy
Pearson r = -0.20
P = 0.0002
cg18608369|methy
Pearson r = -0.20
P = 0.0003
cg01348733|methy
Pearson r = -0.22
P < 0.0001
cg14380270|methy
Pearson r = -0.22
P < 0.0001
cg26573518|methy
Pearson r = -0.22
P < 0.0001
cg05504685|methy
Pearson r = -0.18
P = 0.0008
cg12581244|methy
Pearson r = -0.070
P = 0.2019
cg18411103|methy
Pearson r = -0.085
P = 0.1179
cg08764758|methy
Pearson r = -0.12
P = 0.0257
cg25054416|methy
cg01723139|methy
Pearson r = 0.66
P < 0.0001
cg18124488|methy
cg22282280|methy
Log2(SLFN11|exp+1)

Figure S2

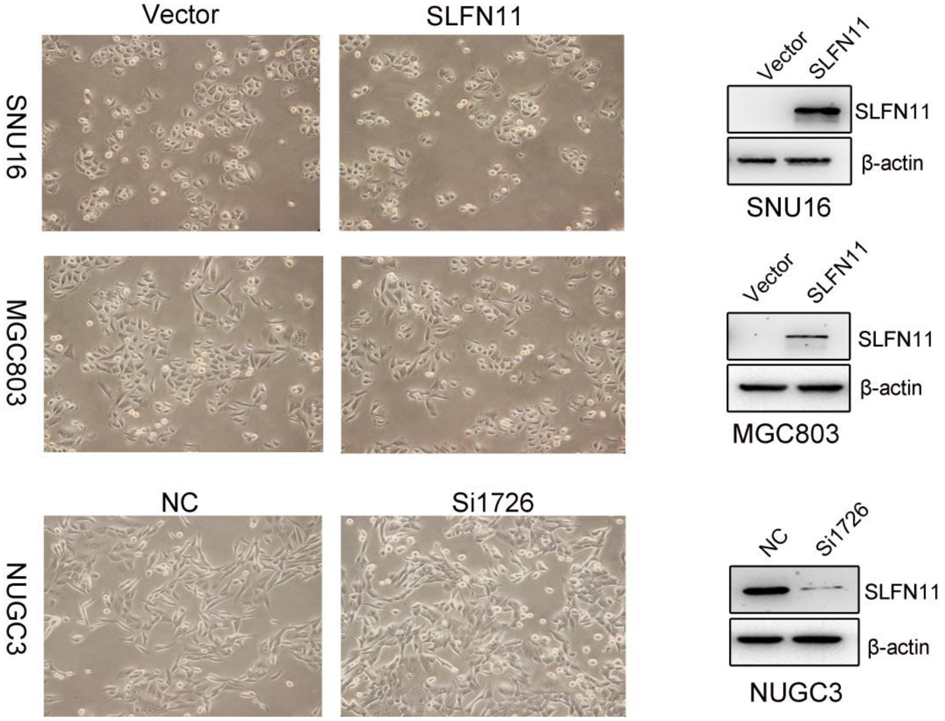

Vector
SLFN11
SNU16
Vector
SLFN11
SLFN11
β-actin
SNU16
MGC803
Vector
SLFN11
SLFN11
β-actin
MGC803
NC
Si1726
NUGC3
NC
Si1726
SLFN11
β-actin
NUGC3
